# A diet based on multiple functional concepts improves cognitive performance in healthy subjects

**DOI:** 10.1186/1743-7075-10-49

**Published:** 2013-07-15

**Authors:** Anne Nilsson, Juscelino Tovar, Maria Johansson, Karl Radeborg, Inger Björck

**Affiliations:** 1 Department of Applied Nutrition and Food Chemistry, Lund University, P.O. Box 124, Lund, SE-221 00, Sweden; 2 Antidiabetic Food Centre, Lund University, Lund, SE-221 00, Sweden; 3 Department of Psychology, Lund University, Lund, Sweden

**Keywords:** Cognitive performance, Cognitive decline, Diet and cognitive functions, Ageing, Metabolic disorders, Metabolic disease, Metabolic syndrome, Dietary prevention, Randomized controlled trial, Crossover design

## Abstract

**Background:**

Disorders such as the metabolic syndrome (MetS), impaired glucose tolerance and diabetes, are associated with increased risk of cognitive decline. Also several of the individual key features that define the MetS, e.g. hypertension, impaired glucose regulation, dyslipidemia, obesity, and inflammation, are related to an increased risk of cognitive decline. Consequently, a diet that prevents metabolic disorders might be expected to prevent cognitive decline. The purpose of the present study was to, in overweight but otherwise healthy subjects, investigate effects on cognitive functions of a dietary regime combining multiple functional concepts potentially beneficial to risk markers associated with MetS. The purpose was in addition to evaluate cognitive performance in relation to results on cardiometabolic risk variables (BMI, blood pressure, glucose, insulin, cholesterol, triglycerides, free fatty acids, lipoprotein A-1 and B, hs-CRP, HbA1c, interleukin-6, TNF-α, and PAI-1).

**Methods:**

Fourty-four healthy women and men (50–73 years, BMI 25–33, fasting glycemia ≤ 6.1 mmol/L) participated in a randomized, controlled crossover intervention, comparing a multifunctional diet (active diet (AD)) including foods with a potential anti-inflammatory action, with a control diet (CD) devoid of the “active” components. Both diets were composed in close agreement with the Nordic dietary recommendations. Each diet was consumed during 4 wk, separated by a 4 wk washout period. Cognitive tests were performed at fasting and in the postprandial period after a standardized breakfast, after each diet period.

**Results:**

In comparison with the CD, the AD improved performance in the Rey Auditory-Verbal Learning test (recognition test, *p* < 0.05, ANOVA, n = 42) and significantly improved performance in test of selective attention, which also included aspects of working memory (*p* < 0.05, n = 40). Performance in cognitive tests was inversely associated with plasma concentrations of cardiometabolic risk markers (fasting cholesterol, blood glucose, blood pressure) and cardiovascular risk scores (Framingham and Reynols), and positivly associated with apolipoprotein A1 (*p* < 0.05).

**Conclusions:**

The results indicate that diet characteristics may modulate cognitive performance. A relationship seems to exist between cardiometabolic risk markers and cognitive performance in apparently healthy subjects. The results provide additional motives for diet based prevention of metabolic disturbances related to the MetS.

## Introduction

Dietary prevention is increasingly recognized as a necessary strategy against the current epidemic of obesity and related metabolic disorders. Epidemiological studies and dietary interventions strongly support that high intake of whole grain foods and legumes are beneficial in the prevention and management of type 2 diabetes (T2DM), management of T1DM [1], and for weight control [2]. Foods with low glycemic index (GI) have further proven beneficial in the prevention and treatment of the metabolic syndrome (MetS), obesity, T2DM, and cardiovascular disease [3-6]. Other dietary measures, such as the inclusion of foods rich in omega-3 fatty acids [7] or polyphenols (e.g. berries) [8], have shown cardioprotective effects by improving blood lipid profile, lowering blood pressure, and positively affecting inflammatory markers [7,9].

Obesity, insulin resistance and T2DM are closely associated with chronic inflammation, predominately in adipose tissue [10,11]. Interestingly, peripheral inflammatory cytokines can be actively transported across the blood–brain-barrier, or induce expression of cytokines in the brain; causing impairment of neuronal function [12,13]. Also, reduced insulin receptor signalling compromizes neuroplasticity; indicative of a link between chronic low grade inflammation, insulin resistance, and cognitive impairment [14,15]. T2DM and the MetS are associated with an increased risk of cognitive decline [16]. Consequently, a diet that prevents metabolic disorders might be expected to prevent an associated cognitive decline.

Certain foods or dietary patterns, e.g. polyphenol rich foods (e.g. berries and cocoa) [17-19], and low GI foods [20,21], may improve cognitive functions and/or prevent cognitive decline. In prospective cohort- [22] and cross-sectional [23] studies of middle aged and elderly populations, higher proportions of n-3 polyunsaturated fatty acids (n-3 PUFA) in plasma were linked to a lower risk of cognitive decline, and a number of studies reveal that higher fish consumption promote better cognitive functions [24-28]. However, few studies have examined the possibilities to beneficially influence cardiometabolic risk markers and measures of cognitive performance by dietary means in apparently healthy subjects. Previously, we reported that an “active” dietary regime, gathering different anti-inflammatory food concepts, markedly reduced acknowledged cardiometabolic risk markers, as well as reduced predicted cardiovascular events in healthy overweighed subjects (BMI, mean ± SEM: 28.5 ± 0.3 kg/m^2^) [29]. The diet was e.g. rich in low glycemic impact meals, antioxidant-rich foods, oily fish and rapeseed oil as sources of omega-3 fatty acids, viscous dietary fibers, soybeans, whole barley kernel products, almonds, stanols, and included also a probiotic strain. The rationale for investigating a healthy cohort was that the overall purpose of the study relates to dietary prevention, which in shorter interventions preferably is evaluated before manifest metabolic diseases have been developed.

The present paper is an extension to the study described above [29], now with the purpose to, in the same cohort, report the impact of the active diet on measures of cognitive functions. The purpose was in addition to evaluate cognitive performance in relation to results on cardiometabolic risk variables (BMI, blood pressure, glucose, insulin, cholesterol, triglycerides, free fatty acids, lipoprotein A-1 and B, hs-CRP, HbA1c, interleukin-6, TNF-α, and PAI-1). Fourty-four overweight but otherwise healthy subjects (50–73 years, BMI 25–33 kg/m^2^) participated in the study (randomized controlled crossover design). The active diet was compared with a control diet devoid of the active components/features. Each diet was consumed during four weeks with a four week washout period in-between.

### Subjects and methods

Information regarding the subjects, diets (active and control), and study protocol for metabolic measurements are thoroughly described elsewhere (J Tovar et. al. 2012 [29]), and just briefly described in this paper. The present paper instead focuses on new data related to the cognitive aspects.

The study was approved by the Regional Ethical Review Board, Lund, Sweden (Dnr 593/2008).

### Subjects

Inclusion criteria were age between 50 and 73 years, BMI 25–33 kg/m^2^, and fasting plasma glucose concentration ≤ 6.1 mmol/L. In total, 44 (36 females, 8 men) healthy subjects between 50 and 73 years of age (mean 63.3 ± 0.8 years) without any known medical condition or cognitive decline were included in the study. The subjects were overweight or slightly obese, but otherwise healthy (BMI (mean ± SEM): 28.5 ± 0.3 kg/m^2^). BMI of two subjects were 25 kg/m^2^, 34 subjects had BMI between 25–30 kg/m^2^, and eight subjects had BMI 30–33 kg/m^2^. More detailed description of subjects characteristics have been presented previously [29].

### Study protocol

The study had a randomized, controlled, balanced crossover design. One active- and one control diet were included in the study (described below). Twenty subjects started with the active diet and 24 subjects with the control diet. Each diet period lasted four weeks, with a four-week washout period in-between. The whole trial included four clinical visits, one before and one after each diet phase. Cognitive tests were performed after each diet period, i.e. at visits no. two and four, whereas metabolic risk markers were determined at fasting at all four visits.

Before each intervention period (visits no. one and three), the participants performed test versions of the Selective Attention (SA) test and got thorough information regarding the Rey Auditory-Verbal Learning test (RAVLT) (the tests are described below)*.* No specific screening for possible cognitive decline was carried out prior to the enrollment. However, performance in the pilot version of the SA-test at visit no. one was taken as a measure of the subjects' cognitive abilities to conduct the study in an adequate manner. In addition the participants underwent a medical examination at the first visit. All participants were considered qualified to participate in the study.

At the cognitive test days (visit no. two and four), the subjects arrived fasting in the morning between 7.30 am and 8.15 am (individually standardized). After resting for 15 minutes in a sitting position, physiological test parameters were determined, including weight, blood pressure, blood glucose, insulin, cholesterol (total cholesterol, LDL (low density lipoprotein), and HDL (high density lipoprotein)), triglycerides, free fatty acids, lipoprotein A-1 and B, hs-CRP (high-sensitivity C-reactive protein), HbA1c (glycated hemoglobin), inflammatory markers (IL-6 (interleukin-6), TNF-α (tumor necrosis factor alpha)), and PAI-1 (plasminogen activator inhibitor 1). A detailed description of the test variables and methods used have been presented previously [29]. The first SA-test was performed after obtaining the fasting metabolic test variables, and thereafter subjects were provided a standardized breakfast consisting of a white wheat bread (75.5 g) (Dollar Storfranska, Lockarps bakery, Malmö, Sweden) and apricot marmalade (27.7 g) (Ica, Sweden), corresponding to 55 g available carbohydrates in total. Water, 100 ml, and a plain cup of decaffeinated coffee or tea (150 ml) were served with the bread. The breakfast was supposed to be consumed within 15 min. Two of the participants were unable to eat the whole bread serving at the first experimental day (visit no. two). The leftovers were weighted and registered, and the subjects were served the same amounts of bread at the second experimental day (visit no. four). The cognitive tests were performed repeatedly up to 120 min after the start of the breakfast. A time schedule for execution of cognitive tests is presented in Table 1.

**Table 1:** Time schedule for execution of cognitive tests

**Time**	**SA-test**	**RAVLT-test**^**2**^
Fasting (0 min)^1^	X	
30 min		
45 min	X	
60 min		X
90 min		
120 min	X	

^1^The first SA-test was performed at fasting, directly prior to a standardised breakfast. ^2^The RAVLT took approximately 60 min to perform. *SA* selective attention, *RAVLT* Rey Auditory Verbal Learning Test.

### Test- and control diets

The diets included (active diet and control diet) were non vegetarian, and designed in close agreement with the Nordic Nutrition Recommendations [30]. The active diet contained foods, food components, and food concepts, with documented potential to reduce sub-clinical inflammation and cardiometabolic risk, such as:

a) foods with high anti-oxidative capacity (anti-inflammatory effects [31], may improve blood pressure and blood lipids [8], suggested to be beneficial to cognitive functions [19]).

b) fatty fish (contain long chain n-3 PUFA, known to have anti-inflammatory and triglyceride-lowering properties [32,33], and are suggested to improve working memory capacity [34]). Rapeseed oil was included to provide additional n-3-PUFA.

c) prebiotic carbohydrates intrinsic in barley kernel products and whole grain rye foods, and products rich in dietary fibre from oat. Included was also bread supplemented with guar gum, which is a highly viscous fibre. Viscous fibre (in barley, oat, and guar gum) may lower the GI, and the indigestible carbohydrates included in the products can have prebiotic effects and improve glycaemic regulation, reduce satiety, and reduce inflammatory markers in a 10-14 h perspective [35,36].

d) Low-GI foods/meals (reduce the risk of T2DM [37] and the MetS [38], reduce inflammatory tonus [39] and may be beneficial to cognitive functions [20,21]).

e) products that improve the blood lipid profile (soybeans, a margarine enriched in stanol esters, and dry almonds [40]).

In addition the active diet included a probiotic strain (*Lactobacillus plantarum* Heal19, DSM 15313) with health benefits on the gut microflora. The control diet contained a similar distribution of energy providing nutrients, but essentially lacked the active food components or food concepts.

The daily energy intake was based on gender, and supplied 2,500-2,600 Kcal/d for men and 2,000-2,100 Kcal/d for women, including foods both from plant and animal origin. The nutritional profiles are presented in Table 2. The mean daily quantities of the different active foods or food components in the active diet, and the main functional properties that were considered for their inclusion, have been summarized in Table 3. The products have been described in more detail previously [29].

**Table 2:** Nutritional profiles of the control and active diet

	**Control diet**		**Active diet**	
	**Women**	**Men**	**Women**	**Men**
Energy (kcal/day)*	2045	2570	2100	2615
Protein (E%)	15	14	19	18
Carbohydrate (E%)	56	55	51	50
Fat (E%)	29	30	31	31
Saturated fat (E%)	12.8	13.2	5.9	5.9
Monounsaturated fat (E%)	10.5	11.1	13.0	13.6
Polyunsaturated fat (E%)	3.6	3.7	8.2	8.4
ω-6 fatty acids (E%)	2.9	3.1	4.2	4.3
ω-3 fatty acids (E%)	0.8	0.8	2.2	2.3
ω-6/w-3 ratio	3.8	3.8	1.9	1.9
Dietary fiber (g/day)	22	26	49	61
Cholesterol (mg/day)	200	240	140	160

* Increased energy intake was prescribed for subjects that experienced weight loss during the initial weeks (Table 2 taken from *Tovar et. al.*[29]).

**Table 3:** Proposed functional action and average content of active components in the active diet

**Active components**	**Main functional role**	**Contents in the AD (g/day)**	
		**Women**	**Men**
Soybean/soy protein	Cholesterol-lowering, anti-inflammatory [40,41]	21	25
VISCOUS FIBERS			
b-glucans	Cholesterol- lowering, prebiotic, GI-reducing [21,42,43]	5.8	6.2
Guar gum		5.6	6.7
Total		11.4	12.9
n-3 PUFA (20:5 + 22:6)	Triglyceride-lowering, anti-inflammatory [32,33]	2.4	3.0
Almonds	Cholesterol-lowering [40,44]	28	28
Plant stanols	Cholesterol-lowering [40,45]	2.0	2.7
Cinnamon	Antioxidant [46]	3.0	3.0
Blueberries	Antioxidant, prebiotic [47]	74.5	94.5
Vinegar	GI –reducing [48]	22.5	22.5
Probiotic	Anti-inflammatory [49]	0.1 (10^10^ CFU)	0.1 (10^10^ CFU)
Whey protein*	GI-reducing [50]	4.3	4.3

*Whey protein (10 g) was only consumed simultaneously with high GI meals (potatoes, parsnip), 3 times per week.

Participants were provided with a 14-day menu plan which was repeated during the last two weeks. They had to follow the recipes in detail for breakfast, lunch, dinner and snacks (cooking procedures, and quantities by e.g. weighing the food ingredients). Since the purpose was that the study would be carried out under a constant weight, subjects had to weigh themselves at home once a week. If there was a discrepancy (up or down) by more than a kg compared with the beginning of a diet period, they were instructed to contact a nutritionist who was attached to the study in order to get help to adjust energy intake. They received a check list to fill in to check off every day that all active food components were consumed, and they had to make a note to describe any deviation from the menu. A limited amount of alcohol (30 and 37 g ethanol/wk for women and men, respectively), was allowed during the intervention period. The volunteers’ ordinary coffee and tea drinking habits were maintained during the trial.

### Cognitive tests

The rationale for the choice of cognitive tests was to include a broad variety of aspect of cognitive functions that are sensitive to metabolic disturbance. The SA-test covers several domains and cognitive aspects, e.g. working memory, attention and choice reaction time. The SA-test is of short duration, slightly less than 10 min, and in this respect making it suitable for repeated testing. We have previously included the SA-test in investigations of food effects on cognitive functions, and investigations of relationships between cognitive functions and metabolic parameters (glucose tolerance), with good results [21,51]. The time points for the SA-test were chosen to cover several aspects with respect to glucose concentrations, and metabolism and its regulation; fasting stage, the postprandial peak increase in blood glucose, and later postprandial phase. The RAVLT took 1 hour to perform, making it difficult to repeat, however word lists have been used extensively with respect to investigations of cognitive effects of metabolic disorders (e.g. Hassenstab JJ et. al 2010 [52]).

#### Selective attention (SA)

The test was computerized, based on spatial perception, and primarily measured the ability to sustain a prolonged attention, and to control and split the attention to the entire picture on the computer screen [21,51]. The SA-test also included aspects of the working memory i.e. simultaneous temporary storing and processing of information. The SA was measured using a test made up of 96 pictures, each shown for two seconds on the screen. The pictures consisted of a square on a white background, divided into four smaller squares. One of the smaller squares was red, one was green, and two squares were uncolored (white), resulting in a total of 12 unique picture combinations. The subjects had to remember the positions of the colored squares, and to compare each new picture that emerged on the screen with the preceding one. Each time a new picture emerged, either the green, the red or none of the colored squares were positioned in the same position compared with the previous picture. Within two seconds, the subjects were supposed to indicate by pressing one of three different keys on the keyboard, which of the three possible alternatives that occurred for each new picture. The SA-test was performed at fasting and at 45, and 120 minutes after start of the standardized breakfast. The SA-test began with a short training session, and took approximately 10 min to perform. The test was scored with the number of correct responses (CR, total 95 credits) and for the reaction time (RT) needed to give the response (i.e. press one of the keys).

#### The Rey Auditory-Verbal Learning test (RAVLT)

The RAVLT [53] was used to measure aspects of learning and memory. The test was modified to avoid ceiling effects. A list of 30 common nouns (originally 15), were read to the subjects, separated by 2 seconds, in 5 consecutive trials. Each reading of a list was followed by an immediate free recall where the nouns were recalled and written down on paper within 2 minutes. Results after the 5th reading measured best learning, and the mean of trials no. 1–5 was a measure of mean learning. In a 6th trial, an interference list of 30 new common nouns were presented, follow by an immediate recall (measuring proactive interference (PI) from list 1). In a 7th trial, the subjects were supposed to recall the first list, without a new reading (measuring retroactive interference (RI) from list 2). After a 20 minute break, in trial no. 8, without an additional reading, the subjects were asked to again recall the first list (delayed recall). The next trial (trial no. 9) was a recognition test. The subjects were given a list with 100 nouns (modified from 50) of which 30 were from the first list, 30 from the second list, and 40 were new nouns. They were asked to, within 3 minutes, identify the 30 words that appeared on the first list. In the last trial (no. 10), the first list was presented on a paper in a different order than the original list (list no. one). They were asked to mark which words (10 nouns) that appeared fist on the original list, which words that appeared in the middle of the list (10 nouns) and which were in the end of the list (10 nouns) (memory of temporal order The test was scored for correct recalled nouns in trials no. 1–8, mean learning (mean of trials no. 1–5), best learning (trial no, 5), recognition (trial no.9), and memory of temporal order (trial no. 10). The total RAVLT took approximately 60 minutes to perform.

### Calculations and statistical methods

A power calculation was performed based on cardiometabolic test markers (plasma LDL-cholesterol and hs-CRP) in the parallel study [29]. Based on this power calculation it was decided to include 44 subjects. This is close to the number of subjects (40 subjects) that have been included in previous meal studies and interventions, using a corresponding SA-test [21,34]. The results are expressed as means ± SEM. The influence of the active- and control diets on the cognitive tests was analyzed by repeated measures ANOVA at the test points, with order of diets, time point of the test, and diets as independent variables, and performance in cognitive tests as dependent variables. Statistical calculations were performed in Stat View 5.0 and SuperAnova 1.11. Significant differences in cognitive performance depending on diet sequence (cognitive test day one or two) were investigate with ANOVA (general linear model), in MINITAB Statistical Software (release 14; Minitab, Minitab Inc, State College, PA). Participants acted as their own control.

Multiple linear regression analysis was performed to examine relationships between cardiometabolic risk markers (independent variables) and performance in cognitive tests (dependent outcome variables), using STATA software package (release 10). A large number of cardiometabolic risk markers were determined (described above). To lower the number of risk markers, and to determine and avoid multicollinearity, pair-wise relationships were examined prior to regression analyses, using Pearson correlations in MINITAB Statistical Software (release 14; Minitab, Minitab Inc, State College, PA). Cardiometabolic risk markers with no tendency to be related with cognitive performance were eliminated. Pearson correlations in all possible pair-wise combinations of the cardiometabolic risk markers were performed. Five cardiometabolic risk markers (systolic blood pressure and fasting concentrations of: total cholesterol, insulin, glucose, ApoA1) were identified as non-linearly combined and included into the multiple regression models (*model no. 1*)*.* Further backward stepwise analysis was conducted by checking the significance of the cardiometabolic risk markers and including in the model only those which were statistically significant (*model 2*). Furthermore, model 1 and model 2 were adjusted for age of the test subjects (*model 3* and *model 4*, respectively). All models were tested for the normality of residuals. Data were tested for outliers, and no extreme or influential data were identified. In the case of a skewed distribution, variables were transformed accordingly before further analyses were performed (SA-tests: correct responses, and RAVLT: mean of trial 1–5 and trial 8 after both active- and control diet, and trial 7 after control diet, were analysed as their square values, trial no. 6 were analysed as there square roots, and cubic transformation were applied for trial no. 9 after both the active- and control diet). Regression analyses were based on data obtained from cognitive tests and risk markers determined at the same visits, generating separate regression analyses after the active diet and control diet, respectively.

Ten-year coronary heart disease risk was calculated with the Framingham Study equation [54], considering age, gender, total cholesterol, HDL-cholesterol, smoking and systolic BP values, and the Reynolds Risk Score [55], which also incorporates CRP values. Relations between Framingham- and Reynolds Risk Score and cognitive performance were evaluated with pairwise Pearson correlations.

Statistical evaluations of cardiometabolic risk variables are described elsewhere [29]. Values of *P* < 0.05 were considered statistically significant.

## Results

Two female subjects failed to accomplish the RAVLT, resulting in n = 42 in this test (23 subjects started with the control diet and 19 subjects with the active diet). Two female subjects did not participate in the SA-test, resulting in n = 42 in the SA-test (22 subjects started with the control diet and 20 subjects with the active diet). Two additional female subjects dropped out from SA-test no. 1 and SA-test no. 3 (n = 40, 21 subjects started with the control diet and 19 subjects with the active diet).

### Effects of diet on cognitive performance

#### SA-TESTS

No main effect depending on diet was observed in the SA-test when all test points were included in the statistical calculation. However, the results revealed significant time effects and interactions (see below) making it relevant to investigate performance in the SA-test at the different time points. Four weeks consumption of the active diet improved performance at 120 min in the SA-test (CR) in comparison with the control diet (Table 4) (*P* < 0.05, ANOVA, n = 40). No significant differences in RT were detected (Table 5).

**Table 4:** Correct responses in the SA-test after 4 weeks consumption of the active- versus the control diet

	**SA-test**^**1**^	
	**Active diet**	**Control diet**
time 0	67.3 ± 2.5	65.6 ± 2.5
45 min	73.4 ± 2.4	74.0 ± 2.1
120 min	76.5 ± 2.4	73.1 ± 2.5*

^1^ SA-test: selective attention test, max 95 credits.

*: *p* < 0.05 for the differences in performance after the active diet in comparison to after the control diet at 120 min. Data are given as means per treatment ± SEM, time = 0: n = 40, time = 45: n = 42, time = 120: n = 40.

**Table 5:** **Reaction times in the SA-test after 4 weeks consumption of the active- versus the control diet **^**1**^

	**SA-test (reaction time (msec)**	
	**Active diet**	**Control diet**
time 0	1320 ± 20	1320 ± 21
45 min	1219 ± 19	1230 ± 21
120 min	1182 ± 21	1202 ± 23

^1^ SA-test: selective attention test.

Data are given as means per treatment ± SEM, time = 0: n = 40, time = 45: n = 42, time = 120: n = 40.

There was a significant time effect in the SA-test, showing improved performance with time at the test days, revealing increased CR at 45 min (73.7 ± 1.6 credits) and 120 min (74.8 ± 1.7 credits) compared with at fasting (66.5 ± 1.8 credits) (*P* < 0.001), and decreased RT (1321 ± 14, 1224 ± 14, and 1190 ± 15 msec at time 0, 45, and 120 min respectively, (*P* < 0.001). There were no main effects in performance of SA-tests (correct responses) depending on the consumption sequence of the diets (i.e. start with the active diet and consume the control diet in the second period (AC group, n = 20), or vice-versa, starting with the control diet and consume the active diet in the last diet period (CA group, n = 24). However, there was a significant diet*consumption sequence interaction (*P* < 0.001), revealing superior performance at all test points after the active diet compared with the control diet, when the active diet was consumed in the second diet period (SA-test 0 min: CR: *P* < 0.001, RT: *P* < 0.01; 45 min: CR and RT: *P* < 0.01, and 120 min: CR: *P* < 0.001, RT: *P* < 0.01). The mean of tests 0–120 min in the CA group was 77.1 ± 2.6 and 69.3 ± 2.7 credits and 1210 ± 23 and 1283 ± 20 msec after the active- and control diet, respectively. In the AC group, superior performance was achieved at 0 min and 45 min after the control diet, when control diet was consumed in the last diet period (SA-test 0 min: CR: *P* < 0.001, RT: *P* < 0.05; 45 min: CR: *P* < 0.01 RT: *P* < 0.05). The mean of tests 0–45 min in the AC group was 72.6 ± 3.8 and 67.2 ± 3.8 credits and 1213 ± 32 and 1273 ± 27 msec after the control- and active diet, respectively. At 120 min in the AC group there was a significant improved RT after the control diet (1154 ± 32 and 1209 ± 34 msec after the control- and active diet, respectively, *P* < 0.05), but no differences in CR depending on diet (71.5 ± 4.1 and 73.4 ± 4.1 credit after active- and control diet respectively, *P* > 0.05).

#### RAVLT

The trial in RAVLT measuring recognition (trial no. 9) was significantly improved after the active diet, in comparison with the control diet (*P* < 0.05, ANOVA, n = 42), (Table 6).

**Table 6:** **Performance in the** RAVLT **after 4 weeks consumption of the active- versus the control diet **^**1**^

**RAVLT, trials no. 1-10**		
	**Active diet**	**Control diet**
1: List 1	12.0 ± 0.73	11.3 ± 0.64
2: List 1	16.8 ± 0.73	15.9 ± 0.80
3: List 1	20.1 ± 0.72	20.0 ± 0.75
4: List 1	21.8 ± 0.71	21.8 ± 0.77
5: List 1 (best learning)	22.7 ± 0.68	22.9 ± 0.74
1-5: List 1 (mean learning 1–5)	18.6 ± 0.65	18.4 ± 0.69
6: List 2 (PI)	9.3 ± 0.62	9.6 ± 0.70
7: List 1 (RI)	21.0 ± 0.80	20.7 ± 0.86
8: 20 min pause. List 1	21.7 ± 0.80	21.7 ± 0.95
9: List 3 (Recognition)	28.0 ± 0.51	27.2 ± 0.69*
10: List 1 (Order of listed nouns)	17.1 ± 0.64	16.0 ± 0.59

^1^ RAVLT: The Rey Auditory-Verbal Learning test.

*: *p* < 0.05 for the differences in performance in RAVLT no. 9 after the active diet in comparison to after the control diet. Data are given as means per treatment ± SEM, n = 42. *PI* proactive interference, *RI* retroactive interference.

#### Effect of test occasion (cognitive experimental day one or two)

Improved performances were observed at the second cognitive test day compared with the first test day in the SA-tests (mean test no. 1–3), both in CR and RT (CR 68.3 ± 2.3 and 74.8 ±2.2 credits at the first and second cognitive experimental day, respectively, P < 0.05; and RT: 1278 ± 16.1 and 1211 ± 18.7 msec at the first and second cognitive experimental day, respectively, P < 0.05).

In the RAVLT, subjects performed significantly better in trial no. 1–5 (mean) at the second test day in comparison to the first test day (trial 1–5 (mean value): 18.0 ± 0.7 and 19.4 ± 0.6 at the first and second cognitive experimental day, respectively, P < 0.05). A similar effect of test day was seen in trials no. 7 (trial 7: firs test day 20.4 ± 0.8 and second test day 21.6 ± 0.8, P < 0.05).

### Relations between cognitive performance and metabolic risk markers

As previously have been reported [29], several metabolic risk markers were improved after four weeks intervention with the active diet, whereas no improvements were seen after the control diet. Briefly, the active diet significantly improved total serum cholesterol (*P* < 0.0001), LDL-cholesterol (*P* < 0.0001), triglycerides (*P* < 0.01), LDL/HDL-ratio (*P* < 0.0001, apoB/apoA1-ratio (*P* < 0.0001), HbA1c (*P* < 0.01), hs-CRP (*P* < 0.05), and systolic blood pressure (*P* < 0.05). In addition, the Framingham and the Reynolds cardiovascular risk scores were both significantly reduced (*P* < 0.0001) after the active diet, with no change after the control diet [29]. Multiple regression analyses revealed that performance on the cognitive tests reported in this manuscript was significantly associated with several of the cardiometabolic risk markers; both after the active diet and the control diet (model 1 and 2, Tables 7, 8 and 9). Regression analyses (model 2) revealed that the number of CR in the SA-tests after both the active- and the control diet were inversely associated with concentrations of total cholesterol (P < 0.05, Table 7). The RT was positively related to total cholesterol (after the active diet, P < 0.05) and systolic blood pressure (after active diet (P < 0.01) and control diet (P < 0.05)). Adjusting for age in the SA-tests (CR and RT) did not affect relationships between metabolic risk markers and cognitive performance after the active diet, however, after the control diet age was a stronger predictor regarding number of CR (model 4: Coef. -131, Std- Err. 54.7, P < 0.05, beta −0.35) than total cholesterol (ns when adjusted for age). After the control diet both age (model 4: Coef. 9.2, Std. Err. 3.2, P < 0.01, beta 0.39) and systolic blood pressure (model 4: Coef. 2.5, Std. Err. 0.99, P < 0.05, beta 0.34) were positively related to the RT. The strongest predictors to performance in the RAVLT after the active diet were systolic blood pressure (P < 0.05-0.01) and glucose concentrations (P < 0.05) (inverse relationship), and Apo A-1 (positive relationship, P < 0.05-0.01), Table 8. Concentration of Apo-A1 was also the strongest predictor to performance in the RAVLT after the control diet (P < 0.05-0.001, Table 9). Further adjusting for age did not affect the results after the active- or the control diet, with the exception that results in RAVLT trial no. 9 tended to be stronger associated with Apo A1 (model 4: Coef. 10604, Std. Err. 4420, P < 0.05, beta 0.34) than glucose concentrations (ns), that was the case without adjustment for age. Framingham- and the Reynolds cardiovascular risk scores to estimate 10-year cardiovascular risk were both highly correlated with performance in nearly all cognitive tests (Tables 10 and 11).

**Table 7:** **Associations between performance in the SA-test**^**1 **^**(CR and RT)**^**2 **^**and cardio-metabolic risk markers after the active- and the control diet, respectively, obtained with regression analyses (model 1 and model 2)**^**3**^

**SA-test**												
	**Active diet**						**Control diet**					
	CR			RT			CR			RT		
MODEL 1:	R^2^: 0.28			R^2^: 0.35			R^2^: 0.21			R^2^: 0.29		
	Adj- R^2^: 0.17			Adj- R^2^: 0.26			Adj- R^2^: 0.11			Adj- R^2^: 0.19		
	P < 0.05			P < 0.01			P = 0.1			P < 0.05		
MODEL 2:	coef	(std Err)	beta	coef	(std Err)	beta	coef	(std Err)	beta	coef	(std Err)	beta
Total- cholesterol	−1048*	(416)	−0.37	53*	(22)	0.32	−535*	(244)	−0.33	-		
ApoA1	-			-			-			-		
Systolic BP	-			2.9**	(0.97)	0.40	-			2.6*	(1.1)	0.36
Glucose	-			-			-			-		
Const.	**9968**	(1812)		**638**	(153)		**8344**	(1453)		**898**	(144)	

^1^*SA* selective attention. ^2^*CR* correct responses, *RT* reaction time.

^3^Cardio-metabolic risk markers included in model 1 are: systolic blood pressure, fasting concentrations of total cholesterol, Apo A-1, insulin, and glucose. Model 2 includes test variables revealing significant associations with cognitive performance after step-wise, one-by-one elimination of non-significant test markers in model 1.

*: *p* < 0.05, **: *p* < 0.01. n = 42.

**Table 8:** **Associations between performance in the RAVL (trials no. 1–10) and cardio-metabolic risk markers after the active diet, obtained with regression analyses (model 1 and model 2) **^**1**^

**RAVLT, active diet**																		
	**Mean trial no. 1–5 (n = 42)**			**Trial no. 6 (n = 40)**			**Trial no. 7 (n = 41)**			**Trial no. 8 (n = 41)**			**Trial no. 9 (n = 41)**			**Trial no. 10 (n = 39)**		
MODEL 1.	R^2^: 0.32			R^2^: 0.31			R^2^: 0.30			R^2^: 0.30			R^2^: 0.24			R^2^: 0.17		
	Adj- R^2^: 0.22			Adj- R^2^: 0.21			Adj- R^2^: 0.21			Adj- R^2^: 0.20			Adj- R^2^: 0.14			Adj- R^2^: 0.04		
	P < 0.05			P < 0.05			P < 0.05			P < 0.05			P = 0.07			P = 0.29		
MODEL 2.	coef	(std Err)	beta	coef	(std Err)	beta	coef	(std Err)	beta	coef	(std Err)	beta	coef	(std Err)	beta	coef	(std Err)	beta
Total- cholesterol	-			-			-			-			-			-		
ApoA1	7.5*	(3.1)	0.32	-			9.6*	(3.8)	0.34	445**	(157)	0.30	-			7.9*	(3.3)	0.36
Systolic BP^2^	−0.10**	(0.04)	−0.40	-			−0.11*	(0.04)	−0.37	−3.9*	(1.7)	−0.31	-			-		
Glucose	-			−0.44*	(0.28)	−0.37	-			-			−3576*	(1449)	−0.36	-		
Const.	21.6	(6.6)		5.4	(0.98)		22.5	(8.0)		382	(327)		42288	(7930)		6.0	(4.7)	

*: *p* < 0.05, **: *p* < 0.01. n = 43.

^1^ Cardio-metabolic risk markers included in model 1 are: systolic blood pressure, fasting concentrations of total cholesterol, Apo A-1, insulin, and glucose. Model 2 includes test variables revealing significant effects on cognitive performance after step-wise, one-by-one elimination of non-significant test markers in model 1. ^2^*BP* blood pressure.

**Table 9:** **Associations between performance in the RAVL (trials no. 1–8)**^**1 **^**and cardio-metabolic risk markers after the control diet, obtained with regression analyses (model 1 and model 2)**^**2**^

**RAVLT, control diet**												
	**Mean trial no. 1–5 (n = 43)**			**Trial no. 6 (n = 41)**			**Trial no. 7 (n = 42)**			**Trial no. 8 (n = 42)**		
MODEL 1.	R^2^: 0.18			R^2^: 0.19			R^2^: 0.30			R^2^: 0.35		
	Adj- R^2^: 0.16			Adj- R^2^: 0.06			Adj- R^2^: 0.21			Adj- R^2^: 0.25		
	P < 0.01			P = 0.21			P < 0.05			P < 0.01		
MODEL 2.	coef	(std Err)	beta	coef	(std Err)	beta	coef	(std Err)	beta	coef	(std Err)	beta
Total cholesterol	-	-		-			-			-		
ApoA1	333**	(109)	0.43	1.3*	(0.51)	0.37	489**	(142)	0.48	574***	(141)	0.54
Systolic BP^3^	-	-		-			-			-		
Glucose	-	-		-			-			-		
Const.	−143	(169)		1.0	(0.80)		−282	(220)		−363	(217)	

^1^ Performance in trial no 9–10 did not reveal significant effects of metabolic risk markers, and the results are therefore not shown in the table.

^2^ Cardio-metabolic risk markers included in model 1 are systolic blood pressure, fasting concentrations of total cholesterol, Apo A-1, insulin and glucose. Model 2 includes test variables revealing significant effects on cognitive performance after step-wise, one-by-one elimination of non-significant test markers in model 1. ^3^*BP* blood pressure.

*: *p* < 0.05, **: *p* < 0.01, ***: *p* < 0.0001.

**Table 10:** **Pearson correlations between Reynold- and Framingham risk scores and performance in the SA-test (mean of tests no. 1–3)**^**1**^

	**SA-test. mean of tests 1-3**			
**Cardiovascular risk score**	**Correct response**		**Reaction time**	
	**Active diet**	**Control diet**	**Active diet**	**Control diet**
Reynold 10 years risk	−0.19	−0.18	0.32*	0.46**
Framingham 10 years risk	−0.03	0.07	0.16	0.33*

^1^*SA-test* selective attention test.

*: *p* < 0.05, **: *p* < 0.01.

**Table 11:** **Pearson correlations between Reynold- and Framingham risk scores and performance in the** RAVLT **no. 1–9)**^**1**^

	**Trial no.1-5**		**Trial no. 6**		**Trial no. list 7**		**Trial no. list 8**		**Trial no. list 9**	
Cardiovascular risk score	Active diet	Control diet	Active diet	Control diet	Active diet	Control diet	Active diet	Active diet	Active diet	Control diet
Framingham risk score	−0.43**	−0.46**	−0.29^§^	−0.38*	−0.42**	−0.37*	−0.41**	−0.41**	−0.40**	−0.42**
Reynold risk score	−0.55***	−0.55***	−0.33*	−0.37*	−0.48**	−0.48**	−0.52***	−0.52***	−0.43**	−0.46**

^1^ RAVLT: The Rey Auditory-Verbal Learning test 1–9, no significant effects were detected in trial no. 10.

*: *p* < 0.05, **: *p* < 0.01, ***: *p* < 0.0001, ^§^: *p* < 0.1.

## Discussion

The present study shows that four-week intervention with a diet that significantly improved cardiovascular risk variables also resulted in favorable effects on cognitive functions. Consequently, improved cognitive performance was observed in the SA-test at 120 min post a standardized breakfast, and in the RAVLT at approximately 115 min (trial no. 9; recognition), after four weeks intervention with the “active” diet compared with the control diet. In this context, it must be noted that from a nutritional viewpoint both the active diet and the control diet were good diets, designed in close agreement with the Nordic Nutrition recommendations [30]. The improved performance in the SA-test and RAVLT (trial no. 9; recognition) were revealed late in the test session, indicative of reduced mental fatigue and/or increased learning capacity (the SA-test) during the test day after the active diet compared with the control diet.

Interestingly, in parallel to the improvements in cognitive functioning, the active diet improved several cardiometabolic risk markers connected to the MetS, e.g. lowered cholesterol concentrations, CRP, blood pressure, and improved measures of glucose regulation (HbA1c).

The purpose of this study was to evaluate cognitive effects of a mixed diet possessing metabolic advantageous. Thus, the study design does not allow any conclusions regarding effects of individual food components. However, the major inclusion criteria for the variety of foods and meals in the active diet were to achieve anti-inflammatory properties through several different food mechanisms [29]. One of the underlying purposes with combining several food concepts was to increase the possibility to include realistic amounts of each active food components, hence increasing compliance. When foods are investigated individually there is often a tendency to provide active test products in rather exaggerated amounts.

It is well established that metabolic disorders such as the MetS, impaired glucose tolerance, and T2DM are associated with increased risk of cognitive decline, e.g. decrease in memory and executive functioning [56-58], information processing speed, and attention [16]. Also several of the individual key features that define the MetS, e.g. hypertension, impaired glucose regulation, dyslipidemia, obesity [59], and inflammation, are related to an increased risk of cognitive decline [60]. The results obtained in the present study indicate that cardiometabolic risk markers included in the MetS seem to be related to cognitive performance also in an apparently healthy cohort, suggesting that negative effects on cognitive functions may occur already in an early stage of metabolic deteriorations. Accordingly, the associations obtained in the present study can be described such that higher levels of metabolic risk markers (systolic blood pressure, total cholesterol, blood glucose) were related to worse performance in cognitive tests, meanwhile the opposite relations were obtained between cognitive performance and the potentially protective marker Apolipoprotein A1. Considering the relatively healthy study population, the results are certainly noteworthy. Additional striking results concerning relationships between cognitive performance and risk of cardiometabolic events, were the strong associations seen between cognitive performance and Framingham- and the Reynolds cardiovascular risk scores to estimate 10-year cardiovascular risk, which both were highly correlated (Pearson correlations) with performance in nearly all cognitive tests (Tables 10 and 11).

The test subjects in the present study can be considered to represent a comparatively healthy cohort. In contrast, other similar diet studies of the age matched population, that is 50–75 years of age, typically include a hyperlipidemic study population [40,61,62]. A recent study revealed that the prevalence of the MetS in the Swedish population is between 10 to 16% among 50-year-old women, 16 to 26% among 50-year-old men, and from 20 to 35% among the 60-year-old men [63]. It was further revealed that only 5% of that population group had no risk factors related to the MetS. In the presently reported study, fifty-nine percent of the volunteers exhibited no MetS-feature, and only one could be classified as MetS-positive. Although it is widely recognized that T2DM and the metabolic syndrome is associated with an increased risk of cognitive decline, the results obtained in this study points towards a tight connection between metabolism and cognitive function also in a healthy study population. The results suggest the possibility of reversing cognitive decline by choice of diet, and highlight the importance of early dietary prevention. The dietary prevention strategy should preferably include dietary patterns aiming at lower sub-clinical inflammation and that beneficially affect several metabolic risk markers simultaneously; which was the purpose of the present study.

Due to the demographic profile of the population, the age-related socioeconomic burden of brain diseases, with or without dementia, is expected to markedly increase during the next two decades. Dementia constitutes a considerable part of these pathologies [64,65]. Of importance in this context is that the increased prevalence of cardiometabolic disorders, such as T2DM and MetS, can be expected to further increase the number of people suffering from cognitive decline. Several lifestyle characteristics, where diet plays an important role, are involved in the age related cognitive decline and dementia. For example, a diet rich in fat, especially of saturated type, has in longitudinal population-based studies (21 y [27] and 6 y [66] follow up), showed to be associated with poorer performance on a variety of cognitive tasks. Fat induced cognitive decline is probably mediated by increased insulin resistance [67]. On the contrary, prospective cohort studies indicate that diets rich in specific foods, such as e.g. fish [25], fruits [68], and polyphenol rich berries [69], may prevent age related cognitive decline.

In older adults with T2DM, it was noted that pharmacological interventions targeting glycemic control may improve aspects of cognitive functions, e.g. working memory, and improvements in cognitive tests were correlated with improvement in glycemic control [70]. Furthermore, in a two-year lifestyle intervention in T2DM with associated cognitive impairment, some aspects of cognitive function were improved to the same level as people with normal glucose tolerance. The intervention aimed to improve glucose control by including group exercise 2–4 times a week as well as nutritional education with recommendations of caloric intake, carbohydrate intake (55 energy%), total fat intake (less than 30–35 energy%), protein intake (10 to 15 energy%), and dietary fiber intake (at least 30 g / day) [71]. Results obtained in the current study provide indications of the possibility of reversing a downward-sloping trend in cognitive performance by diet intervention also in an apparently healthy cohort.

The present investigation was performed in middle-aged overweight volunteers under weight maintenance condition. However, the active diet was highly satiating, and a good proportion of the participants needed instructions by a nutritionist not to lose weight. Overweight and obesity are highly related to increased cardiovascular risk, suggesting a potential for additional cognitive improvements of the active diet if weight loss had been allowed.

The present study had certain limitations. One potential limitation was a possible practise effect, which was indicated by improved performance in the SA-test and some of the RAVLT at the second cognitive experimental day in comparison to the first experimental day. However, the study had a well-balanced design with respect to order of test diets, which probably eliminated this potential limitation.

## Conclusions

The present study indicates that diet characteristics may modulate cognitive performance, and further, a relationship appears to exist between cardiometabolic risk markers and cognitive performance; also in apparently healthy subjects. The results provide additional motives for diet based prevention of metabolic disturbances related to the metabolic syndrome.

## Abbreviations

AD: Active diet; ApoA1: Apolipoprotein A1; Apo B: Apolipoprotein B; BP: Blood pressure; CD: Control diet; hs-CRP: High sensitivity C-reactive protein; HBA1c: Glycated hemoglobin; HDL: High density lipoprotein; IL-6: Interleukin 6; LDL: Low density lipoprotein; MetS: Metabolic syndrome; PAI-1: Plasminogen activator inhibitor 1; RAVLT: The Rey Auditory-Verbal Learning test; RI: Retroactive interference; SA: Selective attention; T2DM: Type 2 diabetes mellitus; TNF-α: Tumor necrosis factor alpha.

## Competing interests

The authors declare that they have no competing interests.

## Authors’ contributions

AN, IB, JT, and MJ were responsible for the study concept. AN was responsible for the design and coordination of the of the part of the study dealing with cognitive measurements. AN and JT conducted the research. KR and AN had the primary responsibility for statistical calculations, and AN and KR were responsible for the evaluations of the results on cognitive tests. AN and IB had primary responsibility for the final content, but all authors (AN, JT, KR, IB, and MJ) were involved in the writing and evaluation of the paper. All authors read and approved the final manuscript.

## References

[B1] Venn BJ Mann JI Cereal grains, legumes and diabetes Eur J Clin Nutr 2004 58 1443 1461 10.1038/sj.ejcn.1601995 15162131

[B2] O'Neil CE Zanovec M Cho SS Nicklas TA Whole grain and fiber consumption are associated with lower body weight measures in US adults: National Health and Nutrition Examination Survey 1999–2004 Nutr Res 2010 30 815 822 10.1016/j.nutres.2010.10.013 21147364

[B3] Brand-Miller JC Glycemic load and chronic disease Nutr Rev 2003 61 S49 S55 10.1301/nr.2003.may.S49-S55 12828192

[B4] Liu S Willett WC Stampfer MJ Hu FB Franz M Sampson L Hennekens CH Manson JE A prospective study of dietary glycemic load, carbohydrate intake, and risk of coronary heart disease in US women Am J Clin Nutr 2000 71 1455 1461 10837285 10.1093/ajcn/71.6.1455

[B5] Salmeron J Ascherio A Rimm EB Colditz GA Spiegelman D Jenkins DJ Stampfer MJ Wing AL Willett WC Dietary fiber, glycemic load, and risk of NIDDM in men Diabetes Care 1997 20 545 550 10.2337/diacare.20.4.545 9096978

[B6] Salmeron J Manson JE Stampfer MJ Colditz GA Wing AL Willett WC Dietary fiber, glycemic load, and risk of non-insulin-dependent diabetes mellitus in women JAMA 1997 277 472 477 10.1001/jama.1997.03540300040031 9020271

[B7] Ebrahimi M Ghayour-Mobarhan M Rezaiean S Hoseini M Parizade SM Farhoudi F Hosseininezhad SJ Tavallaei S Vejdani A Azimi-Nezhad M Omega-3 fatty acid supplements improve the cardiovascular risk profile of subjects with metabolic syndrome, including markers of inflammation and auto-immunity Acta Cardiol 2009 64 321 327 10.2143/AC.64.3.2038016 19593941

[B8] Erlund I Koli R Alfthan G Marniemi J Puukka P Mustonen P Mattila P Jula A Favorable effects of berry consumption on platelet function, blood pressure, and HDL cholesterol Am J Clin Nutr 2008 87 323 331 18258621 10.1093/ajcn/87.2.323

[B9] Zafra-Stone S Yasmin T Bagchi M Chatterjee A Vinson JA Bagchi D Berry anthocyanins as novel antioxidants in human health and disease prevention Mol Nutr Food Res 2007 51 675 683 10.1002/mnfr.200700002 17533652

[B10] Wellen KE Hotamisligil GS Inflammation, stress, and diabetes J Clin Invest 2005 115 1111 1119 15864338 10.1172/JCI25102 PMC1087185

[B11] Hotamisligil GS Inflammation and metabolic disorders Nature 2006 444 860 867 10.1038/nature05485 17167474

[B12] Banks WA Farr SA La Scola ME Morley JE Intravenous human interleukin-1alpha impairs memory processing in mice: dependence on blood–brain barrier transport into posterior division of the septum J Pharmacol Exp Ther 2001 299 536 541 11602664

[B13] Jang S Johnson RW Can consuming flavonoids restore old microglia to their youthful state? Nutr Rev 2010 68 719 728 10.1111/j.1753-4887.2010.00336.x 21091915 PMC3058829

[B14] Reagan LP Insulin signaling effects on memory and mood Curr Opin Pharmacol 2007 7 633 637 10.1016/j.coph.2007.10.012 18023616 PMC2193628

[B15] Huang CC Lee CC Hsu KS The role of insulin receptor signaling in synaptic plasticity and cognitive function Chang Gung Med J 2010 33 115 125 20438663

[B16] van den Berg E Dekker JM Nijpels G Kessels RP Kappelle LJ de Haan EH Heine RJ Stehouwer CD Biessels GJ Cognitive functioning in elderly persons with type 2 diabetes and metabolic syndrome: the Hoorn study Dement Geriatr Cogn Disord 2008 26 261 269 10.1159/000160959 18841011

[B17] Lamport D Dye L Wightman J Lawton CL The effects of flavonoid and other polyphenol consumption on cognitive performance: a systematic research review of human experimental and epidemiological studies Nutr Aging 2012 1 5 25

[B18] Krikorian R Nash TA Shidler MD Shukitt-Hale B Joseph JA Concord grape juice supplementation improves memory function in older adults with mild cognitive impairment Br J Nutr 2010 103 730 734 10.1017/S0007114509992364 20028599

[B19] Krikorian R Shidler MD Nash TA Kalt W Vinqvist-Tymchuk MR Shukitt-Hale B Joseph JA Blueberry supplementation improves memory in older adults J Agric Food Chem 2010 58 3996 4000 10.1021/jf9029332 20047325 PMC2850944

[B20] Benton D Ruffin MP Lassel T Nabb S Messaoudi M Vinoy S Desor D Lang V The delivery rate of dietary carbohydrates affects cognitive performance in both rats and humans Psychopharmacology (Berl) 2003 166 86 90 12488949 10.1007/s00213-002-1334-5

[B21] Nilsson A Radeborg K Bjorck I Effects on cognitive performance of modulating the postprandial blood glucose profile at breakfast Eur J Clin Nutr 2012 66 1039 1043 10.1038/ejcn.2012.80 22781020

[B22] Beydoun MA Kaufman JS Satia JA Rosamond W Folsom AR Plasma n-3 fatty acids and the risk of cognitive decline in older adults: the Atherosclerosis Risk in Communities Study Am J Clin Nutr 2007 85 1103 1111 17413112 10.1093/ajcn/85.4.1103

[B23] Dullemeijer C Durga J Brouwer IA van de Rest O Kok FJ Brummer RJ van Boxtel MP Verhoef P n 3 fatty acid proportions in plasma and cognitive performance in older adults Am J Clin Nutr 2007 86 1479 1485 17991662 10.1093/ajcn/86.5.1479

[B24] Dangour AD Allen E Elbourne D Fletcher A Richards M Uauy R Fish consumption and cognitive function among older people in the UK: baseline data from the OPAL study J Nutr Health Aging 2009 13 198 202 10.1007/s12603-009-0057-2 19262951 PMC12876379

[B25] Morris MC Evans DA Tangney CC Bienias JL Wilson RS Fish consumption and cognitive decline with age in a large community study Arch Neurol 2005 62 1849 1853 10.1001/archneur.62.12.noc50161 16216930

[B26] van Gelder BM Tijhuis M Kalmijn S Kromhout D Fish consumption, n-3 fatty acids, and subsequent 5-y cognitive decline in elderly men: the Zutphen Elderly Study Am J Clin Nutr 2007 85 1142 1147 17413117 10.1093/ajcn/85.4.1142

[B27] Eskelinen MH Ngandu T Helkala EL Tuomilehto J Nissinen A Soininen H Kivipelto M Fat intake at midlife and cognitive impairment later in life: a population-based CAIDE study Int J Geriatr Psychiatry 2008 23 741 747 10.1002/gps.1969 18188871

[B28] Nurk E Drevon CA Refsum H Solvoll K Vollset SE Nygard O Nygaard HA Engedal K Tell GS Smith AD Cognitive performance among the elderly and dietary fish intake: the Hordaland Health Study Am J Clin Nutr 2007 86 1470 1478 17991661 10.1093/ajcn/86.5.1470

[B29] Tovar J Nilsson A Johansson M Ekesbo R Aberg AM Johansson U Bjorck I A diet based on multiple functional concepts improves cardiometabolic risk parameters in healthy subjects Nutr Metab (Lond) 2012 9 29 10.1186/1743-7075-9-29 22472183 PMC3361470

[B30] Nordic Council of Ministers Nutrition Recommendations 2004: integrating nutrition and physical activity 2004 4 Copenhagen: Nordic Council of Ministers

[B31] Valtuena S Pellegrini N Franzini L Bianchi MA Ardigo D Del Rio D Piatti P Scazzina F Zavaroni I Brighenti F Food selection based on total antioxidant capacity can modify antioxidant intake, systemic inflammation, and liver function without altering markers of oxidative stress Am J Clin Nutr 2008 87 1290 1297 18469252 10.1093/ajcn/87.5.1290

[B32] Calder PC n-3 polyunsaturated fatty acids, inflammation, and inflammatory diseases Am J Clin Nutr 2006 83 1505S 1519S 16841861 10.1093/ajcn/83.6.1505S

[B33] Skulas-Ray AC Kris-Etherton PM Harris WS Vanden Heuvel JP Wagner PR West SG Dose–response effects of omega-3 fatty acids on triglycerides, inflammation, and endothelial function in healthy persons with moderate hypertriglyceridemia Am J Clin Nutr 2011 93 243 252 10.3945/ajcn.110.003871 21159789 PMC3138218

[B34] Nilsson A Radeborg K Salo I Bjorck I Effects of supplementation with n-3 polyunsaturated fatty acids on cognitive performance and cardiometabolic risk markers in healthy 51 to 72 years old subjects: a randomized controlled cross-over study Nutr J 2012 11 99 10.1186/1475-2891-11-99 23173831 PMC3564898

[B35] Nilsson A Granfeldt Y Östman E Preston T Bjorck I Effects of GI and content of indigestible carbohydrates of cereal-based evening meals on glucose tolerance at a subsequent standardised breakfast Eur J Clin Nutr 2006 60 1092 1099 10.1038/sj.ejcn.1602423 16523203

[B36] Nilsson AC Östman EM Holst JJ Bjorck IM Including indigestible carbohydrates in the evening meal of healthy subjects improves glucose tolerance, lowers inflammatory markers, and increases satiety after a subsequent standardized breakfast J Nutr 2008 138 732 739 18356328 10.1093/jn/138.4.732

[B37] Dong JY Zhang L Zhang YH Qin LQ Dietary glycaemic index and glycaemic load in relation to the risk of type 2 diabetes: a meta-analysis of prospective cohort studies Br J Nutr 2011 106 1649 1654 10.1017/S000711451100540X 22017823

[B38] McKeown NM Meigs JB Liu S Saltzman E Wilson PW Jacques PF Carbohydrate nutrition, insulin resistance, and the prevalence of the metabolic syndrome in the Framingham Offspring Cohort Diabetes Care 2004 27 538 546 10.2337/diacare.27.2.538 14747241

[B39] Ceriello A Davidson J Hanefeld M Leiter L Monnier L Owens D Tajima N Tuomilehto J Postprandial hyperglycaemia and cardiovascular complications of diabetes: an update Nutr Metab Cardiovasc Dis 2006 16 453 456 10.1016/j.numecd.2006.05.006 16934443

[B40] Jenkins DJ Kendall CW Faulkner D Vidgen E Trautwein EA Parker TL Marchie A Koumbridis G Lapsley KG Josse RG A dietary portfolio approach to cholesterol reduction: combined effects of plant sterols, vegetable proteins, and viscous fibers in hypercholesterolemia Metabolism 2002 51 1596 1604 10.1053/meta.2002.35578 12489074

[B41] Azadbakht L Kimiagar M Mehrabi Y Esmaillzadeh A Hu FB Willett WC Soy consumption, markers of inflammation, and endothelial function: a cross-over study in postmenopausal women with the metabolic syndrome Diabetes Care 2007 30 967 973 10.2337/dc06-2126 17392557

[B42] The US National Cholesterol Education Program, with lots of authors Expert Panel on Detection, Evaluation, and Treatment of High Blood Cholesterol in Adults (2002). Third report of the national cholesterol education program (NCEP) expert panel on detection, evaluation, and treatment of high blood cholesterol in adults (adult treatment panel III) final report Circulation 2002 106 3143 3421 12485966

[B43] Mitsou EK Panopoulou N Turunen K Spiliotis V Kyriacou A Prebiotic potential of barley derived beta-glucan at low intake levels: a randomised, double-blinded, placebo-controlled clinical study A Food Res Int 2010 43 1086 1092 10.1016/j.foodres.2010.01.020

[B44] Griel AE Kris-Etherton PM Tree nuts and the lipid profile: a review of clinical studies Br J Nutr 2006 96 Suppl 2 S68 78 17125536 10.1017/bjn20061866

[B45] Esposito K Giugliano D Mediterranean diet and the metabolic syndrome: the end of the beginning Metab Syndr Relat Disord 2010 8 197 200 10.1089/met.2009.0095 20158447

[B46] Dugoua JJ Seely D Perri D Cooley K Forelli T Mills E Koren G From type 2 diabetes to antioxidant activity: a systematic review of the safety and efficacy of common and cassia cinnamon bark Can J Physiol Pharmacol 2007 85 837 847 10.1139/Y07-080 18066129

[B47] Basu A Du M Leyva MJ Sanchez K Betts NM Wu M Aston CE Lyons TJ Blueberries decrease cardiovascular risk factors in obese men and women with metabolic syndrome J Nutr 2010 140 1582 1587 10.3945/jn.110.124701 20660279 PMC2924596

[B48] Leeman M Ostman E Bjorck I Vinegar dressing and cold storage of potatoes lowers postprandial glycaemic and insulinaemic responses in healthy subjects Eur J Clin Nutr 2005 59 1266 1271 10.1038/sj.ejcn.1602238 16034360

[B49] Lomax AR Calder PC Probiotics, immune function, infection and inflammation: a review of the evidence from studies conducted in humans Curr Pharm Des 2009 15 1428 1518 10.2174/138161209788168155 19442167

[B50] Nilsson M Holst JJ Bjorck IM Metabolic effects of amino acid mixtures and whey protein in healthy subjects: studies using glucose-equivalent drinks Am J Clin Nutr 2007 85 996 1004 17413098 10.1093/ajcn/85.4.996

[B51] Nilsson A Radeborg K Bjorck I Effects of differences in postprandial glycaemia on cognitive functions in healthy middle-aged subjects Eur J Clin Nutr 2009 63 113 120 10.1038/sj.ejcn.1602900 17851459

[B52] Hassenstab JJ Sweat V Bruehl H Convit A Metabolic syndrome is associated with learning and recall impairment in middle age Dement Geriatr Cogn Disord 2010 29 356 362 10.1159/000296071 20424454 PMC2889255

[B53] Vaisman N Voet H Akivis A Vakil E Effect of breakfast timing on the cognitive functions of elementary school students Arch Pediatr Adolesc Med 1996 150 1089 1092 10.1001/archpedi.1996.02170350091016 8859144

[B54] Anderson KM Wilson PW Odell PM Kannel WB An updated coronary risk profile. A statement for health professionals Circulation 1991 83 356 362 10.1161/01.CIR.83.1.356 1984895

[B55] Ridker PM Paynter NP Rifai N Gaziano JM Cook NR C-reactive protein and parental history improve global cardiovascular risk prediction: the Reynolds Risk Score for men Circulation 2008 118 2243 2251 2244p following 2251 10.1161/CIRCULATIONAHA.108.814251 18997194 PMC2752381

[B56] Ruis C Biessels GJ Gorter KJ van den Donk M Kappelle LJ Rutten GE Cognition in the early stage of type 2 diabetes Diabetes Care 2009 32 1261 1265 10.2337/dc08-2143 19366968 PMC2699741

[B57] Cavalieri M Ropele S Petrovic K Pluta-Fuerst A Homayoon N Enzinger C Grazer A Katschnig P Schwingenschuh P Berghold A Schmidt R Metabolic syndrome, brain magnetic resonance imaging, and cognition Diabetes Care 2010 33 2489 2495 10.2337/dc10-0851 20852031 PMC2992176

[B58] Lamport DJ Lawton CL Mansfield MW Dye L Impairments in glucose tolerance can have a negative impact on cognitive function: a systematic research review Neurosci Biobehav Rev 2009 33 394 413 10.1016/j.neubiorev.2008.10.008 19026680

[B59] Taylor VH MacQueen GM Cognitive dysfunction associated with metabolic syndrome Obes Rev 2007 8 409 418 10.1111/j.1467-789X.2007.00401.x 17716298

[B60] Sparkman NL Buchanan JB Heyen JR Chen J Beverly JL Johnson RW Interleukin-6 facilitates lipopolysaccharide-induced disruption in working memory and expression of other proinflammatory cytokines in hippocampal neuronal cell layers J Neurosci 2006 26 10709 10716 10.1523/JNEUROSCI.3376-06.2006 17050710 PMC6674759

[B61] Jenkins DJ Jones PJ Lamarche B Kendall CW Faulkner D Cermakova L Gigleux I Ramprasath V de Souza R Ireland C Effect of a dietary portfolio of cholesterol-lowering foods given at 2 levels of intensity of dietary advice on serum lipids in hyperlipidemia: a randomized controlled trial JAMA 2011 306 831 839 10.1001/jama.2011.1202 21862744

[B62] Adamsson V Reumark A Fredriksson IB Hammarstrom E Vessby B Johansson G Riserus U Effects of a healthy Nordic diet on cardiovascular risk factors in hypercholesterolaemic subjects: a randomized controlled trial (NORDIET) J Intern Med 2011 269 150 159 10.1111/j.1365-2796.2010.02290.x 20964740

[B63] Welin L Adlerberth A Caidahl K Eriksson H Hansson PO Johansson S Rosengren A Svardsudd K Welin C Wilhelmsen L Prevalence of cardiovascular risk factors and the metabolic syndrome in middle-aged men and women in Gothenburg, Sweden BMC Public Health 2008 8 403 10.1186/1471-2458-8-403 19063738 PMC2621201

[B64] Pugliatti M Sobocki P Beghi E Pini S Cassano GB Altamura AC Pozzoli S Rosati G Cost of disorders of the brain in Italy Neurol Sci 2008 29 99 107 18483707 10.1007/s10072-008-0868-7

[B65] Sillanpaa M Andlin-Sobocki P Lonnqvist J Costs of brain disorders in Finland Acta Neurol Scand 2008 117 167 172 10.1111/j.1600-0404.2007.00973.x 18081913

[B66] Morris MC Evans DA Bienias JL Tangney CC Wilson RS Dietary fat intake and 6-year cognitive change in an older biracial community population Neurology 2004 62 1573 1579 10.1212/01.WNL.0000123250.82849.B6 15136684

[B67] Greenwood CE Winocur G High-fat diets, insulin resistance and declining cognitive function Neurobiol Aging 2005 26 Suppl 1 42 45 16257476 10.1016/j.neurobiolaging.2005.08.017

[B68] Loef M Walach H Fruit, vegetables and prevention of cognitive decline or dementia: a systematic review of cohort studies J Nutr Health Aging 2012 16 626 630 10.1007/s12603-012-0097-x 22836704 PMC12878179

[B69] Devore EE Kang JH Breteler MM Grodstein F Dietary intakes of berries and flavonoids in relation to cognitive decline Ann Neurol 2012 72 135 143 10.1002/ana.23594 22535616 PMC3582325

[B70] Ryan CM Freed MI Rood JA Cobitz AR Waterhouse BR Strachan MW Improving metabolic control leads to better working memory in adults with type 2 diabetes Diabetes Care 2006 29 345 351 10.2337/diacare.29.02.06.dc05-1626 16443885

[B71] Yamamoto N Yamanaka G Takasugi E Ishikawa M Yamanaka T Murakami S Hanafusa T Matsubayashi K Otsuka K Lifestyle intervention reversed cognitive function in aged people with diabetes mellitus: two-year follow up Diabetes Res Clin Pract 2009 85 343 346 10.1016/j.diabres.2009.05.014 19520452

